# Ongoing challenges in pulmonary fibrosis and insights from the nintedanib clinical programme

**DOI:** 10.1186/s12931-019-1269-6

**Published:** 2020-01-06

**Authors:** Claudia Valenzuela, Sebastiano Emanuele Torrisi, Nicolas Kahn, Manuel Quaresma, Susanne Stowasser, Michael Kreuter

**Affiliations:** 1 Hospital Universitario de La Princesa, Instituto de Investigación Princesa, Universidad Autónoma de Madrid, Madrid, Spain; 2 University Hospital Policlinico-Vittorio Emanuele, Catania, Italy; 3 Center for Interstitial and Rare Lung Diseases, Thoraxklinik, University of Heidelberg, Heidelberg, Germany; 4 Translational Lung Research Center, Member of the German Center for Lung Research, Heidelberg, Germany; 5 Boehringer Ingelheim International GmbH, Ingelheim am Rhein, Germany

**Keywords:** nintedanib, idiopathic pulmonary fibrosis, literature review, challenges

## Abstract

The approvals of nintedanib and pirfenidone changed the treatment paradigm in idiopathic pulmonary fibrosis (IPF), and increased our understanding of the underlying disease mechanisms. Nonetheless, many challenges and unmet needs remain in the management of patients with IPF and other progressive fibrosing interstitial lung diseases.

This review describes how the nintedanib clinical programme has helped to address some of these challenges. Data from this programme have informed changes to the IPF diagnostic guidelines, the timing of treatment initiation, and the assessment of disease progression. The use of nintedanib to treat patients with advanced lung function impairment, concomitant emphysema, patients awaiting lung transplantation and patients with IPF and lung cancer is discussed. The long-term use of nintedanib and an up-to-date summary of nintedanib in clinical practice are discussed. Directions for future research, namely emerging therapeutic options, precision medicine and other progressive fibrosing interstitial lung diseases, are described.

Further developments in these areas should continue to improve patient outcomes.

## Introduction

**Fig. 1:**
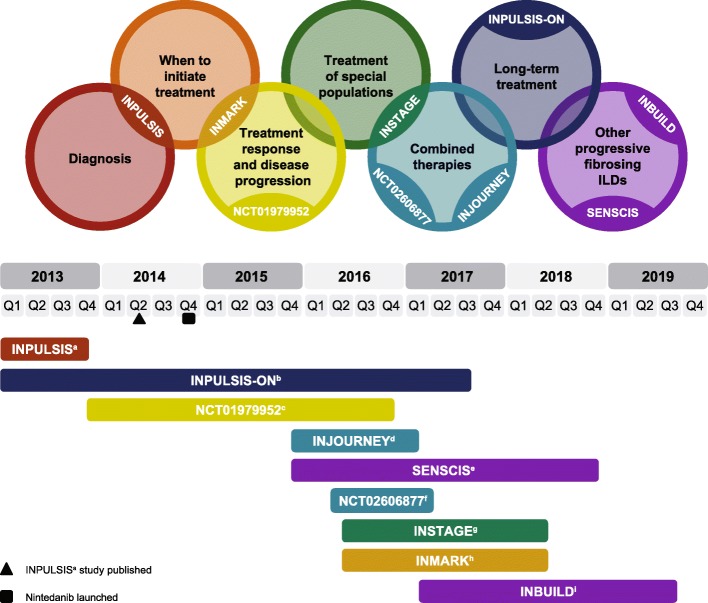
Ongoing challenges and unmet needs in the management of pulmonary fibrosis, and timeline of the nintedanib clinical programme to date. Except where noted, trial duration is depicted as time from enrolment of the first participant until the last visit of the last participant. a, trial of nintedanib versus placebo, shown as time from January 2013 until the last visit of the last participant [2]; b, trial of open-label nintedanib in patients who completed INPULSIS, shown as time from January 2013 until completion of data collection for the primary endpoint analysis [6]; c, trial of nintedanib versus placebo, to examine the effects of nintedanib on quantitative lung fibrosis score [7]; d, trial of add-on pirfenidone versus placebo in patients already receiving nintedanib [8]; e, trial of nintedanib versus placebo in patients with systemic sclerosis-associated interstitial lung disease [9]; f, trial to examine possible pharmacokinetic interactions between nintedanib and pirfenidone [10]; g, trial of sildenafil and nintedanib versus nintedanib alone in patients with advanced lung function impairment [11]; h, trial of nintedanib versus placebo, to examine the effects of nintedanib on concentrations of blood biomarkers for idiopathic pulmonary fibrosis [12]; i, trial of nintedanib versus placebo in patients with progressive fibrosing interstitial lung disease [13]

Idiopathic pulmonary fibrosis (IPF) is a chronic, progressive interstitial lung disease (ILD) of unknown cause in which patients experience worsening lung function resulting from progressive fibrosis. IPF is associated with high rates of morbidity and mortality [1]. Two pharmacological therapies (nintedanib and pirfenidone) have been shown to slow decline in lung function in patients with IPF [1–5]. The United States Food and Drug Administration and European Medicines Agency approvals of nintedanib and pirfenidone changed the treatment paradigm in IPF and increased understanding of the underlying disease mechanisms [5]. However, these approvals raised new questions in the management of ILD, and prior unmet needs remain to be addressed. Such needs can be observed in the following areas: diagnosis; timing of treatment initiation; assessment of treatment response and disease progression; treatment of special patient populations; long-term treatment; and management of other forms of progressive fibrosing ILD (Fig. 1). This review will discuss these challenges in the context of data from the nintedanib clinical programme (Phase II, III and IV clinical trials) and from routine clinical practice in real-world settings (observational cohorts including registries).

## Diagnosis

In the 2011 ATS/ERS/JRS/ALAT guidelines for IPF diagnosis and treatment, the diagnosis of IPF required exclusion of other potential causes of pulmonary fibrosis, and identification of a usual interstitial pneumonia (UIP) pattern by high-resolution computed tomography (HRCT). If a UIP pattern was not clearly identified (“possible” or “inconsistent with UIP”) by HRCT, confirmation of diagnosis via surgical lung biopsy (SLB) was recommended [14]. SLB is associated with an elevated risk of acute exacerbation, pneumothorax and mortality, and may therefore be inappropriate for some patients [15–18].

Reported rates of SLB usage in clinical practice vary. Of patients (N = 417) in the Australian IPF registry diagnosed with IPF, 15% underwent SLB, but 16.5% had radiological features inconsistent with UIP and did not undergo SLB to confirm their diagnosis [19]. In a study of US claims data for patients over 65 years of age with a diagnosis code for IPF, 2518 patients had claims for diagnostic tests for IPF, of which 2.3% had claims for SLB [20]. SLB was used in 26.5% of patients with IPF in the National IPF Registry in Spain, and in 34.1% of patients in the INSIGHTS-IPF registry in Germany [21, 22]. Data from a Europe-wide registry showed that SLB was used in 32% of patients in 2009, but only in 8% of patients in 2016, a decrease attributed to increased use of transbronchial cryobiopsy (cTBB) [23].

An analysis of computed tomography scans and lung biopsies from multiple centres and trials examined the HRCT category of “possible UIP”, defined in the 2011 guidelines, and divided it into “probable UIP” and “indeterminate UIP” (Table 1). In a cohort analysis of pulmonary fibrosis, patients with “probable UIP” by HRCT were significantly more likely to have “probable” or “definite UIP” by SLB than those with “indeterminate UIP” by HRCT [24]. This suggests that patients with “probable” and “indeterminate UIP” should not be grouped together [24]. Furthermore, a *post-hoc* analysis of the INPULSIS trials found that patients with a clinical diagnosis of IPF who had not undergone SLB, but who had traction bronchiectasis without honeycombing by HRCT (similar to patients with “probable UIP” categorized above), had a similar disease course and response to nintedanib as those with honeycombing by HRCT or UIP confirmed by SLB [25]. Altogether, these data suggest that there exists a group of patients within the radiological “possible UIP” group, recommended for SLB according to the 2011 guidelines, whose SLB is likely to confirm a UIP pattern and thus a diagnosis of IPF, and who experience similar disease course and response to treatment as patients with confirmed diagnoses of IPF.

**Table 1:** Summary of studies contributing to change in IPF diagnostic guidelines

Study author, reference	Patient subgroup	HRCT results	SLB results	Diagnosis by 2011 guidelines [14]	Notes
Cohort study involving 201 patients with pulmonary fibrosis who underwent lung biopsy within 1 year of chest CT scan					
Chung [24]	Probable UIP*	Reticulation, little or no honeycombing	Definite*/probable* UIP 82%	IPF	Probable UIP* by HRCT was more likely to have UIP confirmed by SLB than indeterminate UIP* by HRCT
			Indeterminate*18%	Probable IPF	
	Indeterminate for UIP*	Indeterminate	Definite*/probable* UIP 54%	IPF	
			Indeterminate*46%	Probable IPF	
*Post-hoc* analysis of pooled data from the INPULSIS trials on 1061 patients with honeycombing and/or diagnosis of UIP by SLB					
Raghu [25]	Honeycombing or SLB	Honeycombing	Not specified	IPF	Disease progression & response to nintedanib similar between groups
		Not specified	UIP		
	No honeycombing or SLB	Features of possible UIP and traction bronchiectasis, no honeycombing	None available	SLB required	

^*^Definite UIP: peripheral and basilar predominant pulmonary fibrosis characterized by reticulation, honeycombing, and absence of findings to suggest another specific diagnosis; probable UIP: peripheral and basilar predominant pulmonary fibrosis with reticulation, little/no honeycombing but with otherwise typical features of UIP; indeterminate UIP: pulmonary fibrosis with imaging findings not sufficient to reach a definite, probable, or inconsistent with UIP diagnosis [24]

*CT* Computed tomography, *HRCT* High-resolution computed tomography, *IPF* Idiopathic pulmonary fibrosis, *SLB* Surgical lung biopsy, *UIP* Usual interstitial pneumonia

These studies, and others, led to the definition of a “probable UIP” category in the Fleischner Society White Paper and in the updated ATS/ERS/JRS/ALAT diagnosis guidelines, both published in 2018. The 2018 guidelines include a conditional recommendation for SLB in patients with “probable UIP;” the Fleischner Society White Paper discusses that SLB may be unnecessary in these patients, depending on clinical context [26–28].

The 2018 ATS/ERS/JRS/ALAT guidelines note that, for patients with substantial physiological impairment or comorbidities, SLB may have an unfavourable benefit/risk ratio [27]. cTBB is potentially associated with less morbidity and mortality than SLB, and may be more appropriate than SLB for some patients in experienced centres [26, 27, 29, 30]. A real-world study in patients (N = 109) with ILD found no instances of mortality or acute exacerbation within 90 days following cTBB, and that 73.4% of the histological samples obtained had clear diagnostic patterns [31]. A multicentre study of patients (N = 65) with ILD in Australia who each underwent both cTBB and SLB found that the histopathology was consistent in 70.8% of cases. Multidisciplinary diagnosis using samples obtained via cTBB or SLB agreed in 76.9% of cases [32, 33]. However, a smaller study (N = 21) suggested that, although 81% of cTBB samples had diagnostic patterns, concordance between patterns in cTBB and SLB samples may be low [34]. All three studies noted that multidisciplinary discussions were necessary to obtain diagnoses, and that histology was only part of the evidence that contributed to IPF diagnosis [31, 34]. The lack of a standardized procedure for cTBB and the paucity of evidence from large prospective trials means that SLB remains the recommended procedure for most patients [26, 27].

In addition to imaging and histological tests, other procedures can assist in the diagnosis of IPF. Analysis of the composition of bronchoalveolar lavage fluid can help in the diagnostic work-up of suspected IPF, specifically to exclude alternative diagnoses. Serological testing, particularly for antinuclear antibodies, rheumatoid factor, myositis panel and anticyclic citrullinated peptide levels can specifically help in the differential diagnoses of ILDs associated with connective tissue disorders [26, 27].

## When to initiate treatment

The 2015 ATS/ERS/JRS/ALAT IPF treatment guidelines contain conditional recommendations for nintedanib and pirfenidone, but make no suggestions regarding timing of treatment initiation [35]. Real-world data suggest that many patients are not treated with approved IPF therapies immediately after diagnosis, despite the insidious, progressive nature of IPF. In a 2016 European patient chart survey, 53.6% of patients with IPF (N = 1783) were not treated with nintedanib or pirfenidone [36]. A retrospective review of Finnish (n = 158) and Swedish (n = 174) patients with IPF found that, from 2014 to 2016, 45 (29.6%) Finnish and 111 (69.4%) Swedish patients were prescribed nintedanib or pirfenidone [37]. Both studies suggested that patients with higher forced vital capacity (FVC) were less likely to receive antifibrotic treatment [36, 37].

In a physician survey, approximately 50% of responders cited reasons such as “stable” or “asymptomatic” disease, or “good” lung function, for waiting and observing patients before prescribing antifibrotic therapy, and 23% thought that the adverse effects of treatment outweighed the benefits in patients with preserved lung function [38]. Despite this, one observational study of patients in the UK receiving nintedanib found that nintedanib discontinuation rates were lowest in patients with higher FVC (≥ 80%) [39].

**Fig. 2:**
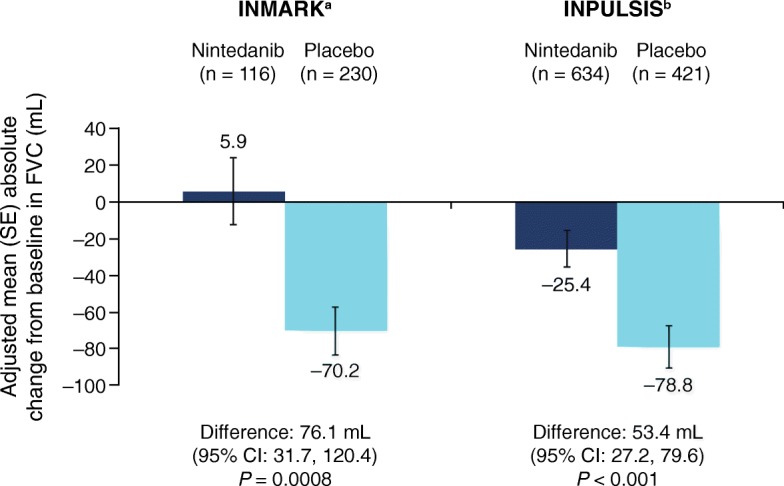
Rate of change in FVC over 12 weeks in the INMARK and INPULSIS trials. a [43],; b [2, 41], and data on file (Boehringer Ingelheim). CI, confidence interval; FVC, forced vital capacity; SE, standard error

Clinical data regarding the efficacy of nintedanib in patients with preserved FVC have been published. Subgroup analyses of the INPULSIS trials have suggested that the treatment effect of nintedanib is consistent across subgroups of disease severity, defined by FVC (> 70 or ≤ 70% pred) and diffusing capacity for carbon monoxide (DL_CO_) (> 40 or ≤ 40% pred) at baseline [40, 41]. Furthermore, data show that the annual rate of lung function decline is already pronounced in patients with more preserved lung function and is similar between subgroups of patients with FVC > 90% or FVC ≤ 90% at baseline who received placebo in the INPULSIS trials (224.6 and 223.6 mL/year, respectively) [42]. In the recent INMARK trial, patients with preserved FVC at baseline (mean 97.5%) receiving nintedanib had a FVC change of +5.9 mL over 12 weeks, whereas those receiving placebo had a change of −70.2 mL (*P* = 0.0008) over 12 weeks [43]. This aligned with FVC changes observed over 12 weeks in patients with less preserved FVC at baseline (mean 79.6%) in the INPULSIS trials (Fig. 2) [2]. Taken together, these data suggest that the rate of decline in FVC in patients with IPF is not dependent on lung function impairment at baseline and is clinically significant in patients with only limited functional impairment.

The INMARK trial featured a 12-week randomized, placebo-controlled period followed by a 40-week period in which all patients received open-label nintedanib, allowing for the assessment of a 12-week delay in treatment initiation in patients with IPF [12]. Nintedanib significantly reduced lung function decline in the initial 12-week period, and decline in FVC over the 40-week period was similar to that observed in the nintedanib arm of the INPULSIS trials. While the difference in FVC decline after 52 weeks was not significant between groups, the 12-week delay in treatment did not appear to be fully compensated for over the 52-week trial period. The proportions of patients with an absolute FVC decline of ≥ 10% or death over 52 weeks were 25% and 30% in the nintedanib and placebo groups, respectively [43].

An analysis of data from clinical practice in Seoul, South Korea indicated that low FVC was a risk factor for acute exacerbations [44]. *Post-hoc* analyses of the INPULSIS trials, and of the STEP-IPF trial of sildenafil in patients with IPF, indicated that patients with lower FVC are at greater risk of acute exacerbations than those with preserved lung function [40, 45, 46]. In two such analyses, nintedanib was associated with a lower risk of acute exacerbations compared with placebo [45, 47].

Collectively, these data suggest that there is no significant difference in the rate of lung function decline between patients with less impaired lung function and those with more impaired lung function, and that the treatment benefit of nintedanib is consistent irrespective of lung function impairment at baseline. While the 2015 ATS/ERS/JRS/ALAT IPF treatment guidelines contain no recommendations regarding timing of nintedanib or pirfenidone initiation, several other guidelines for IPF do [35]. German guidelines state that antifibrotic therapy should be recommended to symptomatic patients at time of diagnosis [48]. A Swiss position paper suggests proposing treatment to patients with IPF when diagnosis is made, especially for patients who have experienced disease progression [49]. French guidelines recommend treating patients with IPF when the patient is diagnosed [50].

## Assessing disease progression and treatment response

FVC and DL_CO_ are the pulmonary function tests (PFTs) most frequently used to assess disease progression [51]. A *post-hoc* analysis of the INPULSIS trials found that FVC decline over 24 weeks was not predictive of further FVC decline in the following 24 weeks of treatment, reflecting the heterogeneous course of IPF [52]. Similarly, a ≥ 10% decline in FVC after 52 weeks in the INPULSIS trials was not predictive of further FVC decline in the first year of the INPULSIS-ON extension study, although it was associated with higher mortality than an FVC decline of < 10% [52]. Concordant data were reported for the CAPACITY and ASCEND trials of pirfenidone [53]. Most patients who continued treatment with either drug did not have a decline in FVC of ≥ 10%, irrespective of prior FVC declines, supporting the continuation of IPF therapy in patients who experience declines in FVC [52, 53]. These results suggest that changes in FVC do not necessarily reflect response to antifibrotic treatment in individual patients.

Change in disease extent quantified by automated interpretation of HRCT is being evaluated as a method of assessing disease progression, but is not established in clinical practice [51, 54]. A quantitative lung fibrosis (QLF) score, derived from HRCT images, has been developed in patients with systemic sclerosis-associated ILD (SSc-ILD) [55]. Change in QLF score correlates with decline in FVC and DL_CO_ in patients with IPF [56]. Data from a prospective trial suggest that nintedanib treatment is associated with a numerically smaller degree of fibrotic change in lungs, by QLF score [7].

Automated interpretation of lung sounds is another potential measure of disease progression in IPF. “Velcro” crackles at baseline, measured by digital auscultation, are associated with the presence of UIP in patients with ILD [57]. A prospective pilot study found that longitudinal changes in lung sounds were associated with clinical deterioration in patients with IPF [58].

Change in St George's Respiratory Questionnaire (SGRQ) scores is a validated measure of quality of life (QoL) in IPF and was a secondary endpoint in the INPULSIS trials [2]. The change in SGRQ total score from baseline was significantly less (corresponding to less deterioration in QoL) in the nintedanib arm of INPULSIS-2 compared with placebo. However, there was no significant difference in SGRQ score between the nintedanib and placebo arms of INPULSIS-1, nor was a significant difference seen in the pooled analysis of the two trials [2]. The suggested minimum important difference in SGRQ score, based on data from the BUILD-1 trial of bosentan in IPF, is between 5–8 points over 6 months [59]. However, an analysis of data from the INPULSIS trials suggested that changes of 4–11 points over 52 weeks were clinically meaningful, although the authors state that further sensitivity analyses are required [60].

The INMARK trial assessed serum levels of neoepitopes (degradation products of the extracellular collagen matrix that accumulates in the lungs of patients with pulmonary fibrosis) for their prognostic value in patients with IPF [12]. Prior work found that the rates of change of six neoepitopes, including C-reactive protein degraded by matrix metalloproteinases 1 and 8 (CRPM), were associated with disease progression and mortality [61]. The primary endpoint of INMARK was the rate of change of serum CRPM after 12 weeks [12]. While there was no significant difference in the rate of change of CRPM between the nintedanib and placebo arms, rising CRPM levels over 12 weeks (compared with falling or stable CRPM levels) were associated with disease progression over 52 weeks. These results confirmed the association of CRPM with disease progression, but did not show that rates of change in neoepitope concentrations were predictive of treatment response [12, 43]. Similarly, a *post-hoc* analysis of the CAPACITY and ASCEND trials of pirfenidone found that C-C motif ligand 18 was prognostic for disease progression, but found no markers predictive of treatment response [62].

## Treatment of special populations

The efficacy of nintedanib in slowing FVC decline was first suggested by the results of the Phase II TOMORROW study, then confirmed in the two replicate Phase III INPULSIS trials [2, 63]. However, further scrutiny is required in some subgroups of patients because of their exclusion from INPULSIS (patients with advanced lung function impairment), comorbidities (patients with severe concomitant emphysema), or the mechanism of action of nintedanib as an angiogenesis inhibitor (patients awaiting lung transplantation; patients with IPF and lung cancer).

### Patients with advanced lung function impairment

Patients with PFTs indicative of advanced lung function impairment (FVC ≤ 50% or DL_CO_ ≤ 30%) were excluded from the INPULSIS trials, hence the efficacy of nintedanib in these patients was not established upon the drug’s approval [2]. The clinical trial INSTAGE examined the efficacy and safety of nintedanib in combination with sildenafil, compared with nintedanib and placebo, in patients with advanced disease defined by DL_CO_ ≤ 35%. While the trial did not meet its primary endpoint (superiority of the combination versus nintedanib alone in the change from baseline in SGRQ total score), the rate of FVC decline in patients treated with nintedanib over 24 weeks aligned with that of patients treated with nintedanib in the INPULSIS trials, whose lung function was less impaired. No new safety signals were identified in INSTAGE, and the most common adverse event (AE) was diarrhoea [11, 64].

While patients entering the INPULSIS trials were required to have limited lung function impairment, measured by FVC and DL_CO_, no such restrictions applied to patients entering the INPULSIS-ON extension study [6, 65]. Of 731 patients who entered INPULSIS-ON and had baseline FVC measurements, 41 (5.6%) had FVC ≤ 50%, while 690 (94.4%) had FVC > 50%. FVC measurements at week 48 were available in 24 and 558 patients with FVC ≤ 50% and FVC > 50%, respectively. Relative and absolute declines in FVC over 48 weeks did not differ between these groups, and were similar to those observed over 52 weeks in patients treated with nintedanib in the INPULSIS trials [65]. Taken together, the data from INSTAGE and INPULSIS-ON suggest that the efficacy of nintedanib extends to patients with more advanced disease and is similar to that in patients with less functional impairment, and that nintedanib has a manageable safety profile irrespective of baseline PFTs [11, 65]. In the future, trials for particular subgroups of patients, such as those with more advanced disease, should be considered.

A single-centre retrospective study of patients with IPF (N = 186) in Philadelphia, USA, found that patients who received nintedanib (n = 57) in clinical practice had lower mean FVC (66%) and DL_CO_ (35%) than those in the INPULSIS trials (79.8% and 47.4%, respectively); no new safety signals were observed despite this [66]. In a single-centre retrospective study in Budapest, Hungary, patients receiving nintedanib (N = 22) were divided into two subgroups, one with FVC < 50% (n = 10), and one with FVC of 50–60% (n = 12). Median survival did not differ between subgroups (444 and 447 days, respectively). The most common AEs were gastrointestinal and elevated liver enzymes. The authors noted that patients with FVC < 50% represented approximately 10% of the total IPF population of their centre [67]. A recent study in Japan examined the effects of nintedanib in patients (N = 22), 8 of whom had advanced lung function impairment (FVC < 50 or DL_CO_/alveolar volume either <30% pred or unmeasurable), and found that the most common AEs in these patients were diarrhoea and elevated serum aminotransferase levels [68].

Lastly, a single-centre retrospective study in Seoul divided patients (N = 108) with IPF into subgroups of more advanced (FVC < 50% or DL_CO_ < 30%) and less advanced (FVC ≥ 50% or DL_CO_ ≥ 30%) lung function impairment at baseline. Most patients (97.2%) experienced AEs; most frequently diarrhoea (50%) and decreased appetite (45.4%). No new safety signals were identified. FVC decline with nintedanib was similar in both subgroups (−1.4% and −3.5% per year for more and less advanced disease, respectively), and was in turn similar to the rate of decline observed in the INPULSIS trials (−2.8% and −3.1% for INPULSIS 1 & 2, respectively) [2, 69].

### Patients with concomitant emphysema

Patients with IPF and emphysema typically present with more preserved FVC than those with isolated IPF [70, 71]. DL_CO_, conversely, is often lower than in patients with IPF alone, possibly because of an additive effect of IPF and emphysema, and the higher frequency of pulmonary hypertension (PH) associated with IPF and emphysema [71–73]. In addition to altered PFTs at discrete timepoints, longitudinal FVC decline appears lower in patients with emphysema and IPF [70, 71]. Stratification of patients with IPF and emphysema by extent of emphysema according to HRCT was used to show that patients with an extent of emphysema of ≥ 15% experienced significantly less FVC decline over 48 weeks than those with an extent of emphysema of < 15%. This finding suggests that longitudinal FVC decline is not an appropriate measure of disease progression in patients with IPF and an extent of emphysema of ≥ 15% [70].

In contrast with other clinical trials, the presence of emphysema at baseline was not an exclusion criterion in the INPULSIS trials [2]. A *post-hoc* analysis of patients with or without emphysema (assessed by interpretation of HRCT scans by a single expert radiologist) in the INPULSIS trials found no significant between-group differences in FVC decline, change in SGRQ total score from baseline, or the risk of first acute exacerbation. These results suggest that the treatment effect of nintedanib is not affected by the presence of emphysema at baseline [74].

### Patients awaiting lung transplantation

Lung transplantation is recommended for the treatment of IPF in eligible patients [14, 35, 75, 76]. Nintedanib is an inhibitor of vascular endothelial growth factor (VEGF), platelet-derived growth factor and fibroblast growth factor (FGF) pathways [77]. Inhibition of the VEGF pathway has been associated with an increased risk of bleeding events [77–81]. VEGF and FGF are involved in wound healing, and potential disruption of this process warrants consideration in patients undergoing lung transplantation [80, 82].

Concerns regarding the use of nintedanib in patients undergoing lung transplantation have not borne out in clinical practice [83–88]. A single-centre study of patients in Belgium receiving nintedanib (n = 2) or pirfenidone (n = 7) observed no bleeding events or wound healing impairment for 20–39 days post-operation [89]. In a larger, single-centre retrospective study of patients who underwent lung transplantations in Germany (N = 287), 62 patients had IPF, for which 23 were undergoing treatment with pirfenidone and 7 were undergoing treatment with nintedanib. No significant increases in intra-operative blood loss, intra- and post-operative use of blood products, wound healing disorders or anastomotic complications were observed between patients with IPF who received nintedanib or pirfenidone and those who did not [90]. A study of patients with ILD undergoing bilateral lung transplantation in Vienna, Austria or Hannover, Germany found that, of 100 patients diagnosed with IPF, 23 received pirfenidone and 13 received nintedanib within 4 weeks prior to surgery. Such use of nintedanib or pirfenidone was not associated with increases in bleeding events, impaired wound healing, or mortality after a median follow-up of 21 months [91].

### Patients with IPF and lung cancer

IPF is a risk factor for lung cancer [92–94]. The reported prevalence of lung cancer in patients with IPF varies from 3–48%, and it is associated with shorter survival times than IPF alone [94–96]. Nintedanib, in combination with docetaxel, is indicated after first-line therapy for the treatment of non-small-cell lung cancer (NSCLC) with adenocarcinoma tumour histology [97]. Nintedanib is known to hinder angiogenesis, which is essential for tumour growth, metastasis and progression [77, 98]. Nintedanib, therefore, has theoretical potential in the treatment of lung cancer associated with IPF, although the most common histotype of lung cancer in IPF is squamous cell carcinoma, and nintedanib is indicated for adenocarcinoma [93, 94, 97, 98].

Chemotherapy, surgical procedures and radiotherapy are all associated with a high risk of acute exacerbations in patients with IPF and lung cancer. Surgery-related mortality is increased in patients with IPF and lung cancer, compared with patients with lung cancer alone [94, 99].

Evidence for the efficacy of nintedanib in patients with IPF and NSCLC is available from a single case study, in which a nodule later identified as squamous cell carcinoma in a patient with IPF remained stable with nintedanib treatment, but increased in size after discontinuation [100]. The J-SONIC trial is investigating the efficacy of nintedanib (compared with no nintedanib) in patients with NSCLC and IPF who are receiving carboplatin and nanoparticle albumin-bound paclitaxel [101].

## Long-term treatment

The INPULSIS trials showed that nintedanib treatment slows disease progression in patients with IPF over 52 weeks [2]. IPF is a chronic progressive disease that requires treatment for longer than 52 weeks, hence extension studies of the INPULSIS trials (and the Phase II TOMORROW trial) have been conducted [6, 102].

In the INPULSIS-ON extension study, patients (N = 734), who received either placebo (n = 304) or nintedanib (n = 430) in INPULSIS, were treated with open-label nintedanib. The mean exposure to nintedanib in INPULSIS-ON was 31.5 months (range 0.0–56.3); the mean total exposure in INPULSIS and INPULSIS-ON was 44.7 months (11.9–68.3). No new safety signals were identified, and the most common AE was diarrhoea. The overall safety profile of nintedanib over the 4-year INPULSIS-ON extension study was similar to that established in the earlier 52-week INPULSIS trials [6].

In the INPULSIS trials, more patients in the nintedanib groups reported a "myocardial infarction" event than in the placebo groups (2.7% vs 1.2%). Conversely, more patients in the placebo groups reported an "ischaemic disease" event than in the nintedanib group (3.1% vs 1.7%) [103]. This imbalance in reported myocardial infarction was not observed in clinical trials of nintedanib in patients with SSc-ILD (SENSCIS) or in patients with other progressive fibrosing ILDs (INBUILD) [104, 105]. Reported bleeding events in the INPULSIS trials, including epistaxis and contusion, were higher in nintedanib groups than in placebo groups, and serious bleeding events were reported with similar incidence between groups [88, 103]. Rates of major adverse cardiovascular events, myocardial infarction and bleeding events in INPULSIS-ON were similar to or lower than those observed in the INPULSIS trials, suggesting that continued nintedanib treatment is not associated with an increase in the risk of these events [6].

Lung function decline over 192 weeks was assessed as an exploratory endpoint in INPULSIS-ON. The annual rate of FVC decline was 135.1 mL, 145.0 mL in patients who continued nintedanib, and 119.7 mL in patients who initiated nintedanib in INPULSIS-ON [6]. The annual rate of FVC decline in the INPULSIS trials was 113.6 mL for patients receiving nintedanib, and 223.5 mL for patients receiving placebo [2, 6]. This suggests that the treatment benefit of nintedanib could extend beyond 4 years [6].

## Use of nintedanib in real-world clinical practice

Nintedanib is currently authorized in over 70 countries for the treatment of IPF. The estimated cumulative exposure from first approval in October 2014 through end of May 2019 is > 80,000 patient-years [106]. The available real-world data support the safety and efficacy profiles of nintedanib that were established in clinical trials [83, 86, 107–111]. Observational studies have reported declines in FVC of < 5% over 9–11 months in most patients treated with nintedanib [85, 109, 110]. Diarrhoea remains the most commonly reported AE, occurring in 33–73% of patients [39, 66, 68, 83–86, 108–110]. In the INPULSIS trials, 63% of patients who received nintedanib reported diarrhoea, compared with 18% of patients who received placebo [2]. Reported discontinuation rates range from 11–45% [39, 66, 84–87, 109, 110]. No new safety signals or increases in bleeding or cardiovascular events have been reported [39, 66, 83–87, 91, 108–110].

A 2015 systematic review found that the most common comorbidities in patients with IPF were PH, obstructive sleep apnoea, lung cancer, chronic obstructive pulmonary disease (including emphysema), ischaemic heart disease (IHD) and gastro-oesophageal reflux disease (GERD) [95]. A real-world study found that lung cancer, arteriosclerosis, IHD and other cardiovascular diseases were significantly associated with impaired outcome in patients with IPF [112]. With the exception of lung cancer, these comorbidities were also reported in patients in clinical practice who received nintedanib (Table 2). The presence of comorbidities at baseline did not appear to affect the safety or efficacy profile of nintedanib [66, 67, 85, 86, 110]. Similarly, concomitant medications at baseline largely had no significant effects on nintedanib treatment [66, 85, 110].

**Table 2:** Comorbidities and concomitant medication use at baseline in real-world IPF populations receiving nintedanib

Study author, year [reference]	Galli, 2017 [66]	Brunnemer, 2018 [110]	Bonella, 2016 [85]	Barczi, 2019 [67]	Tzouvelekis, 2018 [86]	Kreuter, 2017 [113]
Number of patients	57	64	62	22	94	623
Comorbidities, n (%)						
Arterial hypertension		28 (43.8)	19 (31)	14 (63.6)	41 (43.6)	
PH	11 (19.3)	5 (7.8)		9 (40.9)	16 (17.0)	
Congestive heart failure	4 (7)					
IHD	14 (24.6)	21 (32.8)	8 (13)	≤5 (22.7)^a^	20 (21.3)	
Diabetes mellitus	15 (26.3)	16 (25)	9 (14.5)	4 (18.2)	18 (19.1)	
GERD	31 (54.4)	21 (32.8)	7 (11)	2 (9.1)	38 (40.4)	192 (30.8)
OSA		9 (14.1)	4 (6)			
Emphysema	12 (21.1)	9 (14.1)				55 (8.8)
Stroke		2 (3.1)				
Concomitant medications, n (%)						
Prednisone	10 (17.5)					
Anti-acid therapy	37 (64.9)	22 (34.4) (PPI)	16 (26) (PPI)			
Anticoagulant	7 (12.3)	7 (10.9)	2 (3)			
Aspirin + anticoagulant		3 (4.7)				
*N*-acetyl cysteine			5 (8)			
MMF	5 (8.8)					
Sildenafil/tadalafil	6 (10.5)					
Anti-hypertensive		28 (43.8)	19 (31)			

^a^Included under "cardiovascular diseases" in [67], which also included non-IHD, left heart failure, valvular insufficiency

*GERD* Gastro-oesophageal reflux disease, *IHD* Ischaemic heart disease, *IPF* Idiopathic pulmonary fibrosis, *MMF* Mycophenolate mofetil, *OSA* Obstructive sleep apnoea, *PH* Pulmonary hypertension, *PPI* Proton pump inhibitor

In two case studies an improvement in FVC was observed after initiation of nintedanib following an acute exacerbation [114, 115]. There is a lack of real-world data on post-exacerbation survival in patients treated with nintedanib or pirfenidone. However, data from the European IPF Registry (eurIPFreg), the INSIGHTS-IPF registry (Germany) and the Australian Idiopathic Pulmonary Fibrosis Registry suggest that patients with IPF who receive antifibrotic treatment have better overall survival than patients with IPF who do not receive nintedanib or pirfenidone [23, 116, 117]. *Post-hoc* analyses of the INPULSIS trials observed an association between nintedanib treatment and a numerical reduction in mortality following an acute exacerbation [45, 47].

## Future directions

### Emerging therapeutic strategies

Research into improved treatment options for patients with IPF continues. Several studies investigated combination therapy using nintedanib and pirfenidone. A multicentre prospective study in Japan (N = 50) observed an increase in reports of nausea and vomiting when patients receiving pirfenidone were treated with nintedanib, compared with patients receiving pirfenidone alone [118]. The addition of nintedanib to pirfenidone was not associated with any additional safety signals in a prospective international study of patients (N = 89) on a stable dose of pirfenidone [119]. In the INJOURNEY clinical trial (N = 104), the addition of pirfenidone to nintedanib was associated with an increase in gastrointestinal AEs, compared with nintedanib alone. However, 34 patients (64%) who received combination therapy completed the trial, suggesting that combination treatment is feasible in a large proportion of patients. Exploratory efficacy analysis suggested that combination therapy reduced FVC decline to a greater extent than nintedanib alone [8]. A smaller study (N = 37) observed no pharmacokinetic interactions between the two drugs [10]. Data from large, prospective studies are, however, absent.

No safety signals associated with switching from pirfenidone to nintedanib are evident from real-world data [66, 85, 120]. No difference in efficacy was observed between patients who initiated nintedanib after discontinuing pirfenidone and those who were pirfenidone-naïve. A common reason for pirfenidone discontinuation was disease progression; the efficacy of nintedanib in these patients suggests that patients who experience disease progression under pirfenidone could still benefit from treatment with nintedanib [85, 110]. In a small number of patients (n = 4) who transitioned from nintedanib to pirfenidone, no new safety signals were observed [66].

The aforementioned clinical trial INSTAGE assessed the effect of sildenafil and nintedanib on QoL, compared with nintedanib alone, in patients with advanced lung function impairment. While the addition of sildenafil gave only a numerical improvement in QoL, the results of this trial suggested that sildenafil might confer additional benefit in lowering the risk of disease progression in these patients [11]. Furthermore, the benefit of combination therapy on QoL may not have reached the threshold for significance because the study was underpowered, as the INSTAGE trial was powered under the assumption that the effect of sildenafil on QoL would not be affected by nintedanib therapy. The trial design did not account for the possibility of a more pronounced treatment effect of nintedanib on QoL in patients with lower FVC, as observed in subgroup analyses of the INPULSIS trials [11, 40]. Future clinical trials in patients with IPF and a greater degree of lung function impairment should take these potential design limitations into consideration. The effect of combination therapy was consistent irrespective of the presence of right heart dysfunction at baseline [64]. The treatment guidelines for IPF give a conditional recommendation against sildenafil for the treatment of IPF, but make no recommendation regarding the treatment of patients with PH and IPF [35].

GERD is a common comorbidity in IPF; however, data on the use of anti-acid therapies are conflicting. While prespecified analysis of placebo cohorts from the IPFnet programme suggested positive effects [121], a *post-hoc* analysis of data from the placebo arms of the CAPACITY and ASCEND trials suggested that the use of anti-acid therapies does not affect disease course in patients with IPF, and international guidelines give a conditional recommendation for these therapies in patients with IPF and GERD [35, 121, 122]. A *post-hoc* analysis of the INPULSIS trials found that use of anti-acid therapies (proton pump inhibitors (PPIs) and histamine-2 receptor agonists) at baseline did not affect the treatment benefit of nintedanib [123]. The treatment of GERD with PPIs might be associated with an increased risk of enteric bacterial infection [124], and data suggest that patients with IPF and FVC < 70% pred who receive anti-acid therapies are at greater risk of infections (general and pulmonary) than those who do not [122].

In addition to clinical research using existing therapies, novel pharmacotherapies are in development [125]. As nintedanib and pirfenidone are now considered standard of care in IPF, several trials of novel therapeutics (including GLPG1690 [126], PRM-151 [127], PBI-4050 [128] and pamrevlumab [129]) allowed concomitant therapy with nintedanib or pirfenidone in both treatment and placebo arms [125–129]. During a Phase II trial of PBI-4050, an inhibitor of differentiation of fibroblasts into myofibroblasts, an apparent interaction with pirfenidone was observed. Change in mean FVC from baseline to week 12 was numerically superior in the PBI-4050 plus nintedanib group (+0.06% pred) than in either the PBI-4050 alone (−1.11%) or the PBI-4050 plus pirfenidone (−2.69%) groups [128]. Future combination regimens will, therefore, be dependent on the pharmacokinetic and safety profiles of both agents.

Non-pharmacological interventions are also being explored. A systematic review found that pulmonary rehabilitation can improve exercise capacity and QoL in patients with ILD, including IPF, albeit in the short term [130]. In the SPRINT-IPF trial (NCT03717012), pulmonary rehabilitation in combination with nintedanib will be tested against nintedanib alone in patients with IPF (N = 290, planned). The primary endpoint is the change in 6-minute walk distance [131].

### Precision medicine

Despite the relevance of PFTs in the assessment of disease progression, there is a paucity of biomarkers capable of predicting response to treatment or disease progression in individual patients [12, 43]. The identification of such biomarkers could help address the unmet need to develop endpoints that more accurately reflect the degree of fibrogenesis, matrix turnover and functional consequences of fibrosis [12, 132].

### Other progressive fibrosing ILDs

Progressive pulmonary fibrosis is the hallmark of IPF, but this phenotype occurs in other ILDs [13, 133–136]. In general, treatment for these diseases involves off-label use of corticosteroids and immunosuppressive agents [135]. Due to mechanistic similarities between IPF and progressive fibrosing ILD, nintedanib therapy is under investigation in SSc-ILD and in other progressive fibrosing ILDs [9, 13, 104].

Systemic sclerosis is a rare autoimmune disease characterized by fibrosis of the skin and internal organs. ILD is one of the leading causes of morbidity and mortality in SSc [9, 104, 137]. SSc-ILD has an estimated prevalence of 1.7–4.2 per 100,000 individuals in Europe [138]. The SENSCIS trial investigated the use of nintedanib in patients with SSc-ILD. The primary endpoint was the annual rate of FVC decline, which was −52.4 mL per year in patients receiving nintedanib (n = 288) and −93.3 mL per year in patients receiving placebo (n = 288) [104]. These rates are lower than those in the INPULSIS trials (−113.6 and −223.5 mL per year in the nintedanib and placebo arms, respectively), probably because FVC decline in SSc-ILD follows a more heterogeneous course than the irreversible progressive decline observed in patients with IPF [2, 6, 139–141]. Furthermore, the SENSCIS trial included a heterogeneous patient population, and 48% of patients were receiving continuing treatment with mycophenolate mofetil [104]. The relative reduction in FVC decline associated with nintedanib versus placebo in SENSCIS (44%) was similar to that observed in the INPULSIS trials (49%) [2, 104]. The AE profile of nintedanib in patients with SSc-ILD was similar to that observed in patients with IPF, although a higher proportion of patients reported diarrhoea (76% and 32% in the nintedanib and placebo arms, respectively) in the SENSCIS trial than in the INPULSIS trials (62% and 18% in the nintedanib and placebo arms, respectively). potentially arising from the underlying systemic disease [2, 104].

Nintedanib has also been investigated in patients with ILDs that have developed a progressive fibrosing phenotype. The disease in these patients is characterized by decline in lung function, increasing extent of fibrosis by HRCT, or worsening of respiratory symptoms, despite treatment with immunomodulatory therapies [13, 134]. The INBUILD trial assessed the efficacy and safety of nintedanib in patients with fibrosing ILDs and a progressive phenotype, excluding IPF [13, 142]. The primary endpoint was the annual rate of FVC decline, which was −80.8 mL in patients treated with nintedanib (n = 332), compared with −187.8 mL in patients treated with placebo (n = 331). Inclusion was based on extent of fibrosis by HRCT (≥ 10%) and a common underlying progressive phenotype rather than diagnoses of any particular ILD, and INBUILD therefore included patients with ILDs such as: hypersensitivity pneumonitis (n = 173, 26%); autoimmune-associated ILDs (n = 170, 26%), such as rheumatoid arthritis-associated ILD (n = 89, 13%) and SSc-ILD (n = 39, 6%); idiopathic non-specific interstitial pneumonia (n = 125, 19%); and unclassifiable idiopathic interstitial pneumonia (n = 114, 17%). Patients were stratified by the presence or absence of a UIP-like pattern by HRCT. The annual rates of FVC decline in patients with UIP-like pattern were −82.9 mL and −211.1 mL in patients treated with nintedanib and placebo, respectively. In patients with other HRCT patterns these were −79.0 mL and −154.2 mL, respectively. The most common AE was diarrhoea, which occurred in 67% and 25% of patients receiving nintedanib and placebo, respectively. The efficacy of nintedanib in these patients could suggest common pathobiological mechanisms in fibrosing ILDs, irrespective of clinical diagnosis [142, 143]. A real-world study of patients (N = 11) with fibrosing ILDs that had developed a progressive phenotype who were treated with either pirfenidone (n = 10) or nintedanib (n = 1) observed that antifibrotic therapy was associated with stabilization of FVC, further indicating that this approach might be a valuable treatment option [144].

## Conclusion

While the treatment landscape of IPF is growing increasingly favourable, many challenges and unmet needs remain. The diagnosis of IPF is still complex, but research into new techniques that could improve the sensitivity of diagnosis and reduce the burden of histological procedures is ongoing. Lung function tests are, to date, the best measure of disease progression and, although prediction of disease progression in individual patients using PFTs remains problematic, exploration of quantification of disease progression and treatment response using HRCT, digital lung auscultation and blood biomarkers shows promise. Long-term treatment with nintedanib raised no new safety signals, and suggested that the treatment benefit extends beyond 4 years. Real-world evidence has largely corroborated the safety and efficacy profiles of nintedanib established in clinical trials. The efficacy and safety of nintedanib to treat other progressive fibrosing ILDs have been examined in clinical trials. Further developments in these areas, and in the treatment of patients with advanced lung function impairment, concomitant emphysema and lung cancer should continue to improve patient outcomes.

## Data Availability

Data sharing is not applicable to this article as no datasets were generated or analysed.

## Abbreviations

AE: Adverse event
CI: Confidence interval
CRPM: C-Reactive protein degraded by matrix metalloproteinases 1 and 8
CT: Computed tomography
cTBB: Transbronchial cryobiopsy
DL_CO_: Diffusing capacity of the lungs for carbon monoxide
FGF: Fibroblast growth factor
FVC: Forced vital capacity
GERD: Gastro-oesophageal reflux disease
HRCT: High-resolution computed tomography
IHD: Ischaemic heart disease
ILD: Interstitial lung disease
IPF: Idiopathic pulmonary fibrosis
MMF: Mycophenolate mofetil
NSCLC: Non-small-cell lung cancer
OSA: Obstructive sleep apnoea
PFT: Pulmonary function test
PH: Pulmonary hypertension
PPI: Proton pump inhibitor
QLF: Quantitative lung fibrosis
QoL: Quality of life
SE: Standard error
SGRQ: St George’s Respiratory Questionnaire
SLB: Surgical lung biopsy
SSc-ILD: Systemic sclerosis-associated interstitial lung disease
UIP: Usual interstitial pneumonia
VEGF: Vascular endothelial growth factor

## References

[1] Lederer DJ, Martinez FJ (2018). Idiopathic pulmonary fibrosis. N Engl J Med..

[2] Richeldi L, du Bois RM, Raghu G, Azuma A, Brown KK, Costabel U, Cottin V, Flaherty KR, Hansell DM, Inoue Y, Kim DS, Kolb M, Nicholson AG, Noble PW, Selman M, Taniguchi H, Brun M, Le Maulf F, Girard M, Stowasser S, Schlenker-Herceg R, Disse B, Collard HR (2014). Efficacy and safety of nintedanib in idiopathic pulmonary fibrosis. N Engl J Med..

[3] Noble PW, Albera C, Bradford WZ, Costabel U, Glassberg MK, Kardatzke D, King TE, Lancaster L, Sahn SA, Szwarcberg J, Valeyre D, du Bois RM (2011). Pirfenidone in patients with idiopathic pulmonary fibrosis (CAPACITY): two randomised trials. The Lancet..

[4] King TE, Bradford WZ, Castro-Bernardini S, Fagan EA, Glaspole I, Glassberg MK, Gorina E, Hopkins PM, Kardatzke D, Lancaster L, Lederer DJ, Nathan SD, Pereira CA, Sahn SA, Sussman R, Swigris JJ, Noble PW (2014). A phase 3 trial of pirfenidone in patients with idiopathic pulmonary fibrosis. N Engl J Med..

[5] Rivera-Ortega P, Hayton C, Blaikley J, Leonard C, Chaudhuri N (2018). Nintedanib in the management of idiopathic pulmonary fibrosis: clinical trial evidence and real-world experience. Ther Adv Respir Dis..

[6] Crestani B, Huggins JT, Kaye M, Costabel U, Glaspole I, Ogura T, Song JW, Stansen W, Quaresma M, Stowasser S, Kreuter M (2019). Long-term safety and tolerability of nintedanib in patients with idiopathic pulmonary fibrosis: results from the open-label extension study, INPULSIS-ON. Lancet Respir Med.

[7] Goldin J, Kim GH, Trampisch M, Homik L, Hotchkin DL, Ilowite J, Kaye M, Mogulkoc N, Ryerson CJ, Lancaster L, Conoscenti CS. Effect of nintedanib on quantitative lung fibrosis score in patients with idiopathic pulmonary fibrosis (IPF). Pacific Grove: International Colloquium On Lung And Airway Fibrosis; 19 July 2018; 2018.

[8] Vancheri C, Kreuter M, Richeldi L, Ryerson CJ, Valeyre D, Grutters JC, Wiebe S, Stansen W, Quaresma M, Stowasser S, Wuyts WA (2018). Nintedanib with add-on pirfenidone in idiopathic pulmonary fibrosis. Results of the INJOURNEY trial. Am J Respir Crit Care Med.

[9] Distler O, Brown KK, Distler JHW, Assassi S, Maher TM, Cottin V, Varga J, Coeck C, Gahlemann M, Sauter W, Schmidt H, Highland KB (2017). Design of a randomised, placebo-controlled clinical trial of nintedanib in patients with systemic sclerosis-associated interstitial lung disease (SENSCIS). Clin Exp Rheumatol.

[10] Richeldi L, Fletcher S, Adamali H, Chaudhuri N, Wiebe S, Wind S, Hohl K, Baker A, Schlenker-Herceg R, Stowasser S, Maher TM (2019). No relevant pharmacokinetic drug–drug interaction between nintedanib and pirfenidone. Eur Respir J.

[11] Kolb M, Raghu G, Wells AU, Behr J, Richeldi L, Schinzel B, Quaresma M, Stowasser S, Martinez FJ (2018). Nintedanib plus sildenafil in patients with idiopathic pulmonary fibrosis. N Engl J Med.

[12] Maher TM, Stowasser S, Nishioka Y, White ES, Cottin V, Noth I, Selman M, Blahova Z, Wachtlin D, Diefenbach C, Jenkins RG (2018). Investigating the effects of nintedanib on biomarkers of extracellular matrix turnover in patients with IPF: design of the randomised placebo-controlled INMARK® trial. BMJ Open Respir Res.

[13] Flaherty KR, Brown KK, Wells AU, Clerisme-Beaty E, Collard HR, Cottin V, Devaraj A, Inoue Y, Le Maulf F, Richeldi L, Schmidt H, Walsh S, Mezzanotte W, Schlenker-Herceg R (2017). Design of the PF-ILD trial: a double-blind, randomised, placebo-controlled phase III trial of nintedanib in patients with progressive fibrosing interstitial lung disease. BMJ Open Respir Res.

[14] Raghu G, Collard HR, Egan JJ, Martinez FJ, Behr J, Brown KK, Colby TV, Cordier J-F, Flaherty KR, Lasky JA, Lynch DA, Ryu JH, Swigris JJ, Wells AU, Ancochea J, Bouros D, Carvalho C, Costabel U, Ebina M, Hansell DM, Johkoh T, Kim DS, Talmadge E, King J, Kondoh Y, Myers J, Müller NL, Nicholson AG, Richeldi L, Selman M, Dudden RF, Griss BS, Protzko SL, Schünemann HJ (2011). An official ATS/ERS/JRS/ALAT statement: Idiopathic pulmonary fibrosis: Evidence-based guidelines for diagnosis and management. Am J Respir Crit Care Med.

[15] Cottin V (2016). Lung biopsy in interstitial lung disease: balancing the risk of surgery and diagnostic uncertainty. Eur Respir J.

[16] Hutchinson JP, Fogarty AW, McKeever TM, Hubbard RB (2016). In-hospital mortality after surgical lung biopsy for interstitial lung disease in the United States. 2000 to 2011. Am J Respir Crit Care Med.

[17] Hutchinson JP, McKeever TM, Fogarty AW, Navaratnam V, Hubbard RB (2016). Surgical lung biopsy for the diagnosis of interstitial lung disease in England: 1997–2008. Eur Respir J.

[18] Raj R, Raparia K, Lynch DA, Brown KK (2017). Surgical lung biopsy for interstitial lung diseases. Chest..

[19] Jo HE, Glaspole I, Goh N, Hopkins PMA, Moodley Y, Reynolds PN, Chapman S, Walters EH, Zappala C, Allan H, Macansh S, Grainge C, Keir GJ, Hayen A, Henderson D, Klebe S, Heinze SB, Miller A, Rouse HC, Duhig E, Cooper WA, Mahar AM, Ellis S, McCormack SR, Ng B, Godbolt DB, Corte TJ (2019). Implications of the diagnostic criteria of idiopathic pulmonary fibrosis in clinical practice: Analysis from the Australian Idiopathic Pulmonary Fibrosis Registry. Respirology.

[20] Mortimer K, Hartmann N, Chan C, Norman H, Wallace L, Enger C (2019). Characterizing idiopathic pulmonary fibrosis patients using US Medicare-advantage health plan claims data. BMC Pulm Med.

[21] Fernández-Fabrellas E, Molina-Molina M, Soriano JB, Portal JAR, Ancochea J, Valenzuela C, Xaubet A (2019). Demographic and clinical profile of idiopathic pulmonary fibrosis patients in Spain: the SEPAR National Registry. Respir Res.

[22] Behr J, Kreuter M, Hoeper MM, Wirtz H, Klotsche J, Koschel D, Andreas S, Claussen M, Grohé C, Wilkens H, Randerath W, Skowasch D, Meyer FJ, Kirschner J, Gläser S, Herth FJF, Welte T, Huber RM, Neurohr C, Schwaiblmair M, Kohlhäufl M, Höffken G, Held M, Koch A, Bahmer T, Pittrow D (2015). Management of patients with idiopathic pulmonary fibrosis in clinical practice: the INSIGHTS-IPF registry. Eur Respir J.

[23] Guenther A, Krauss E, Tello S, Wagner J, Paul B, Kuhn S, Maurer O, Heinemann S, Costabel U, Barbero MAN, Müller V, Bonniaud P, Vancheri C, Wells A, Vasakova M, Pesci A, Sofia M, Klepetko W, Seeger W, Drakopanagiotakis F, Crestani B (2018). The European IPF registry (eurIPFreg): baseline characteristics and survival of patients with idiopathic pulmonary fibrosis. Respir Res.

[24] Chung JH, Chawla A, Peljto AL, Cool CD, Groshong SD, Talbert JL, McKean DF, Brown KK, Fingerlin TE, Schwarz MI, Schwartz DA, Lynch DA (2015). CT scan findings of probable usual interstitial pneumonitis have a high predictive value for histologic usual interstitial pneumonitis. Chest.

[25] Raghu G, Wells AU, Nicholson AG, Richeldi L, Flaherty KR, Maulf FL, Stowasser S, Schlenker-Herceg R, Hansell DM (2017). Effect of nintedanib in subgroups of idiopathic pulmonary fibrosis by diagnostic criteria. Am J Respir Crit Care Med.

[26] Lynch DA, Sverzellati N, Travis WD, Brown KK, Colby TV, Galvin JR, Goldin JG, Hansell DM, Inoue Y, Johkoh T, Nicholson AG, Knight SL, Raoof S, Richeldi L, Ryerson CJ, Ryu JH, Wells AU (2018). Diagnostic criteria for idiopathic pulmonary fibrosis: a Fleischner Society White Paper. Lancet Respir Med.

[27] Raghu G, Remy-Jardin M, Myers JL, Richeldi L, Ryerson CJ, Lederer DJ, Behr J, Cottin V, Danoff SK, Morell F, Flaherty KR, Wells A, Martinez FJ, Azuma A, Bice TJ, Bouros D, Brown KK, Collard HR, Duggal A, Galvin L, Inoue Y, Jenkins RG, Johkoh T, Kazerooni EA, Kitaichi M, Knight SL, Mansour G, Nicholson AG, SNJ P, Buendía-Roldán I, Selman M, Travis WD, SLF W, Wilson KC (2018). Diagnosis of Idiopathic pulmonary fibrosis. An official ATS/ERS/JRS/ALAT clinical practice guideline. Am J Respir Crit Care Med.

[28] Raghu Ganesh, Remy-Jardin Martine, Myers Jeffrey, Richeldi Luca, Wilson Kevin C. (2019). The 2018 Diagnosis of Idiopathic Pulmonary Fibrosis Guidelines: Surgical Lung Biopsy for Radiological Pattern of Probable Usual Interstitial Pneumonia Is Not Mandatory. American Journal of Respiratory and Critical Care Medicine.

[29] Hetzel Jürgen, Maldonado Fabien, Ravaglia Claudia, Wells Athol U., Colby Thomas V., Tomassetti Sara, Ryu Jay H., Fruchter Oren, Piciucchi Sara, Dubini Alessandra, Cavazza Alberto, Chilosi Marco, Sverzellati Nicola, Valeyre Dominique, Leduc Dimitri, Walsh Simon L.F., Gasparini Stefano, Hetzel Martin, Hagmeyer Lars, Haentschel Maik, Eberhardt Ralf, Darwiche Kaid, Yarmus Lonny B., Torrego Alfonso, Krishna Ganesh, Shah Pallav L., Annema Jouke T., Herth Felix J.F., Poletti Venerino (2018). Transbronchial Cryobiopsies for the Diagnosis of Diffuse Parenchymal Lung Diseases: Expert Statement from the Cryobiopsy Working Group on Safety and Utility and a Call for Standardization of the Procedure. Respiration.

[30] Tomassetti S, Wells AU, Costabel U, Cavazza A, Colby TV, Rossi G, Sverzellati N, Carloni A, Carretta E, Buccioli M, Tantalocco P, Ravaglia C, Gurioli C, Dubini A, Piciucchi S, Ryu JH, Poletti V (2016). Bronchoscopic lung cryobiopsy increases diagnostic confidence in the multidisciplinary diagnosis of idiopathic pulmonary fibrosis. Am J Respir Crit Care Med.

[31] Wälscher Julia, Groß Benjamin, Eberhardt Ralf, Heussel Claus Peter, Eichinger Monika, Warth Arne, Lasitschka Felix, Herth Felix J.F., Kreuter Michael (2018). Transbronchial Cryobiopsies for Diagnosing Interstitial Lung Disease: Real-Life Experience from a Tertiary Referral Center for Interstitial Lung Disease. Respiration.

[32] Troy LK, Grainge C, Corte TJ, Williamson JP, Vallely MP, Cooper WA, Mahar A, Myers JL, Lai S, Mulyadi E, Torzillo PJ, Phillips MJ, Jo HE, Webster SE, Lin QT, Rhodes JE, Salamonsen M, Wrobel JP, Harris B, Don G, PJC W, Ng BJ, Oldmeadow C, Raghu G, EMT L, Arnold D, Cao C, Cashmore A, Cleary S, Evans T-J, French B, Geis M, Glenn L, Hibbert M, Ing A, James A, Meredith G, Merry C, Pudipeddi A, Saghaie T, Thomas R, Thomson C, Twaddell S. Diagnostic accuracy of transbronchial lung cryobiopsy for interstitial lung disease diagnosis (COLDICE): a prospective, comparative study. Lancet Respir Med. 2019. 10.1016/S2213-2600(19)30342-X 31578168

[33] Troy LK, Grainge C, Corte T, Williamson JP, Vallely MP, Cooper W, Mahar AM, Lai S, Mulyadi E, Torzillo PJ, Salamonsen M, Don G, Myers J, Raghu G, Lau EMT (2019). Cryobiopsy versus open lung biopsy in the diagnosis of interstitial lung disease (COLDICE): protocol of a multicentre study. BMJ Open Respir Res.

[34] Romagnoli Micaela, Colby Thomas V., Berthet Jean-Philippe, Gamez Anne Sophie, Mallet Jean-Pierre, Serre Isabelle, Cancellieri Alessandra, Cavazza Alberto, Solovei Laurence, Dell’Amore Andrea, Dolci Giampiero, Guerrieri Aldo, Reynaud Paul, Bommart Sébastien, Zompatori Maurizio, Dalpiaz Giorgia, Nava Stefano, Trisolini Rocco, Suehs Carey M., Vachier Isabelle, Molinari Nicolas, Bourdin Arnaud (2019). Poor Concordance between Sequential Transbronchial Lung Cryobiopsy and Surgical Lung Biopsy in the Diagnosis of Diffuse Interstitial Lung Diseases. American Journal of Respiratory and Critical Care Medicine.

[35] Raghu G, Rochwerg B, Zhang Y, Garcia CAC, Azuma A, Behr J, Brozek JL, Collard HR, Cunningham W, Homma S, Johkoh T, Martinez FJ, Myers J, Protzko SL, Richeldi L, Rind D, Selman M, Theodore A, Wells AU, Hoogsteden H, Schünemann HJ (2015). An official ATS/ERS/JRS/ALAT clinical practice guideline: Treatment of idiopathic pulmonary fibrosis. An update of the 2011 clinical practice guideline. Am J Respir Crit Care Med.

[36] Maher TM, Molina-Molina M, Russell A-M, Bonella F, Jouneau S, Ripamonti E, Axmann J, Vancheri C (2017). Unmet needs in the treatment of idiopathic pulmonary fibrosis―insights from patient chart review in five European countries. BMC Pulm Med.

[37] Pesonen I, Carlson L, Murgia N, Kaarteenaho R, Sköld CM, Myllärniemi M, Ferrara G (2018). Delay and inequalities in the treatment of idiopathic pulmonary fibrosis: the case of two Nordic countries. Multidiscip Respir Med.

[38] Maher TM, Swigris JJ, Kreuter M, Wijsenbeek M, Cassidy N, Ireland L, Axmann J, Nathan SD (2018). Identifying barriers to idiopathic pulmonary fibrosis treatment: A survey of patient and physician views. Respiration.

[39] Fletcher SV, Jones MG, Renzoni EA, Parfrey H, Hoyles RK, Spinks K, Kokosi M, Kwok A, Warburton C, Titmuss V, Thillai M, Simler N, Maher TM, Brereton CJ, Chua F, Wells AU, Richeldi L, Spencer LG (2018). Safety and tolerability of nintedanib for the treatment of idiopathic pulmonary fibrosis in routine UK clinical practice. ERJ Open Res.

[40] Costabel U, Inoue Y, Richeldi L, Collard HR, Tschoepe I, Stowasser S, Azuma A (2016). Efficacy of nintedanib in idiopathic pulmonary fibrosis across prespecified subgroups in INPULSIS. Am J Respir Crit Care Med..

[41] Brown KK, Flaherty KR, Cottin V, Raghu G, Inoue Y, Azuma A, Huggins JT, Richeldi L, Stowasser S, Stansen W, Schlenker-Herceg R, Maher TM, Wells AU (2019). Lung function outcomes in the INPULSIS trials of nintedanib in idiopathic pulmonary fibrosis. Respir Med.

[42] Kolb M, Richeldi L, Behr J, Maher TM, Tang W, Stowasser S, Hallmann C, du Bois RM (2017). Nintedanib in patients with idiopathic pulmonary fibrosis and preserved lung volume. Thorax.

[43] Maher TM, Stowasser S, Nishioka Y, White ES, Cottin V, Noth I, Selman M, Rohr KB, Michael A, Ittrich C, Diefenbach C, Jenkins RG, on behalf of the INMARK trial investigators (2019). Biomarkers of extracellular matrix turnover in patients with idiopathic pulmonary fibrosis given nintedanib (INMARK study): a randomised, placebo-controlled study. Lancet Respir Med.

[44] Song JW, Hong S-B, Lim C-M, Koh Y, Kim DS (2011). Acute exacerbation of idiopathic pulmonary fibrosis: incidence, risk factors and outcome. Eur Respir J..

[45] Collard HR, Richeldi L, Kim DS, Taniguchi H, Tschoepe I, Luisetti M, Roman J, Tino G, Schlenker-Herceg R, Hallmann C, du Bois RM (2017). Acute exacerbations in the INPULSIS trials of nintedanib in idiopathic pulmonary fibrosis. Eur Respir J.

[46] Collard HR, Yow E, Richeldi L, Anstrom KJ, Glazer C, investigators ftI (2013). Suspected acute exacerbation of idiopathic pulmonary fibrosis as an outcome measure in clinical trials. Respir Res.

[47] Kreuter M, Koegler H, Trampisch M, Geier S, Richeldi L (2019). Differing severities of acute exacerbations of idiopathic pulmonary fibrosis (IPF): insights from the INPULSIS® trials. Respir Res.

[48] Behr J, Günther A, Bonella F, Geißler K, Koschel D, Kreuter M, Prasse A, Schönfeld N, Sitter H, Müller-Quernheim J, Costabel U (2017). S2k-Leitlinie Idiopathische Lungenfibrose – Update zur medikamentösen Therapie 2017. Pneumologie.

[49] Funke-Chambour M, Azzola A, Adler D, Barazzone-Argiroffo C, Benden C, Boehler A, Bridevaux PO, Brutsche M, Clarenbach CF, Hostettler K, Kleiner-Finger R, Nicod LP, Soccal PM, Tamm M, Geiser T, Lazor R (2017). Idiopathic pulmonary fibrosis in Switzerland: Diagnosis and treatment. Respiration.

[50] Cottin V, Crestani B, Cadranel J, Cordier JF, Marchand-Adam S, Prevot G, Wallaert B, Bergot E, Camus P, Dalphin JC, Dromer C, Gomez E, Israel-Biet D, Jouneau S, Kessler R, Marquette CH, Reynaud-Gaubert M, Aguilaniu B, Bonnet D, Carre P, Danel C, Faivre JB, Ferretti G, Just N, Lebargy F, Philippe B, Terrioux P, Thivolet-Bejui F, Trumbic B, Valeyre D (2017). French practical guidelines for the diagnosis and management of idiopathic pulmonary fibrosis - 2017 update. Rev Mal Respir..

[51] Robbie H, Daccord C, Chua F, Devaraj A (2017). Evaluating disease severity in idiopathic pulmonary fibrosis. Eur Respir Rev.

[52] Richeldi L, Crestani B, Azuma A, Kolb M, Selman M, Stansen W, Quaresma M, Stowasser S, Cottin V (2019). Outcomes following decline in forced vital capacity in patients with idiopathic pulmonary fibrosis: Results from the INPULSIS and INPULSIS-ON trials of nintedanib. Respir Med.

[53] Nathan SD, Albera C, Bradford WZ, Costabel U, du Bois RM, Fagan EA, Fishman RS, Glaspole I, Glassberg MK, Glasscock KF, King TE, Lancaster L, Lederer DJ, Lin Z, Pereira CA, Swigris JJ, Valeyre D, Noble PW, Wells AU (2016). Effect of continued treatment with pirfenidone following clinically meaningful declines in forced vital capacity: analysis of data from three phase 3 trials in patients with idiopathic pulmonary fibrosis. Thorax.

[54] Weatherley Nicholas D, Eaden James A, Stewart Neil J, Bartholmai Brian J, Swift Andrew J, Bianchi Stephen Mark, Wild Jim M (2019). Experimental and quantitative imaging techniques in interstitial lung disease. Thorax.

[55] Kim HG, Tashkin DP, Clements PJ, Li G, Brown MS, Elashoff R, Gjertson DW, Abtin F, Lynch DA, Strollo DC, Goldin JG (2010). A computer-aided diagnosis system for quantitative scoring of extent of lung fibrosis in scleroderma patients. Clin Exp Rheumatol.

[56] Kim HJ, Brown MS, Chong D, Gjertson DW, Lu P, Kim HJ, Coy H, Goldin JG (2015). Comparison of the quantitative CT imaging biomarkers of idiopathic pulmonary fibrosis at baseline and early change with an interval of 7 months. Acad Radiol.

[57] Sellarés J, Hernández-González F, Lucena CM, Paradela M, Brito-Zerón P, Prieto-González S, Benegas M, Cuerpo S, Espinosa G, Ramírez J, Sánchez M, Xaubet A (2016). Auscultation of velcro crackles is associated with usual interstitial pneumonia. Medicine.

[58] Sgalla G, Larici AR, Sverzellati N, Bartholmai B, Walsh SLF, Nikolic D, Barney A, Fletcher S, Jones M, Davies DD, Richeldi L (2019). Quantitative analysis of lung sounds for monitoring idiopathic pulmonary fibrosis: a prospective pilot study. Eur Respir J..

[59] Swigris JJ, Brown KK, Behr J, du Bois RM, King TE, Raghu G, Wamboldt FS (2010). The SF-36 and SGRQ: Validity and first look at minimum important differences in IPF. Respir Med..

[60] Swigris JJ, Wilson H, Esser D, Conoscenti CS, Stansen W, Kline Leidy N, Brown KK (2018). Psychometric properties of the St George’s Respiratory Questionnaire in patients with idiopathic pulmonary fibrosis: Insights from the INPULSIS trials. BMJ Open Respir Res.

[61] Jenkins RG, Simpson JK, Saini G, Bentley JH, Russell A-M, Braybrooke R, Molyneaux PL, McKeever TM, Wells AU, Flynn A, Hubbard RB, Leeming DJ, Marshall RP, Karsdal MA, Lukey PT, Maher TM (2015). Longitudinal change in collagen degradation biomarkers in idiopathic pulmonary fibrosis: an analysis from the prospective, multicentre PROFILE study. Lancet Respir Med.

[62] Neighbors M, Cabanski CR, Ramalingam TR, Sheng XR, Tew GW, Gu C, Jia G, Peng K, Ray JM, Ley B, Wolters PJ, Collard HR, Arron JR (2018). Prognostic and predictive biomarkers for patients with idiopathic pulmonary fibrosis treated with pirfenidone: Post-hoc assessment of the CAPACITY and ASCEND trials. Lancet Respir Med.

[63] Richeldi L, Costabel U, Selman M, Kim DS, Hansell DM, Nicholson AG, Brown KK, Flaherty KR, Noble PW, Raghu G, Brun M, Gupta A, Juhel N, Klüglich M, du Bois RM (2011). Efficacy of a tyrosine kinase inhibitor in idiopathic pulmonary fibrosis. N Engl J Med..

[64] Behr Jürgen, Kolb Martin, Song Jin Woo, Luppi Fabrizio, Schinzel Birgit, Stowasser Susanne, Quaresma Manuel, Martinez Fernando J. (2019). Nintedanib and Sildenafil in Patients with Idiopathic Pulmonary Fibrosis and Right Heart Dysfunction. A Prespecified Subgroup Analysis of a Double-Blind Randomized Clinical Trial (INSTAGE). American Journal of Respiratory and Critical Care Medicine.

[65] Wuyts WA, Kolb M, Stowasser S, Stansen W, Huggins JT, Raghu G (2016). First data on efficacy and safety of nintedanib in patients with idiopathic pulmonary fibrosis and forced vital capacity of ≤50 % of predicted value. Lung.

[66] Galli JA, Pandya A, Vega-Olivo M, Dass C, Zhao H, Criner GJ (2017). Pirfenidone and nintedanib for pulmonary fibrosis in clinical practice: Tolerability and adverse drug reactions. Respirology.

[67] Barczi E, Starobinski L, Kolonics-Farkas A, Eszes N, Bohacs A, Vasakova M, Hejduk K, Müller V (2019). Long-term effects and adverse events of nintedanib therapy in idiopathic pulmonary fibrosis patients with functionally advanced disease. Adv Ther.

[68] Nakamura M, Okamoto M, Fujimoto K, Ebata T, Tominaga M, Nouno T, Zaizen Y, Kaieda S, Tsuda T, Kawayama T, Hoshino T (2019). A retrospective study of the tolerability of nintedanib for severe idiopathic pulmonary fibrosis in the real world. Ann Transl Med.

[69] Yoon H-Y, Park S, Kim DS, Song JW (2018). Efficacy and safety of nintedanib in advanced idiopathic pulmonary fibrosis. Respir Res.

[70] Cottin V, Hansell DM, Sverzellati N, Weycker D, Antoniou KM, Atwood M, Oster G, Kirchgaessler K-U, Collard HR, Wells AU (2017). Effect of emphysema extent on serial lung function in patients with idiopathic pulmonary fibrosis. Am J Respir Crit Care Med..

[71] Akagi T, Matsumoto T, Harada T, Tanaka M, Kuraki T, Fujita M, Watanabe K (2009). Coexistent emphysema delays the decrease of vital capacity in idiopathic pulmonary fibrosis. Respir Med.

[72] Seeger W, Adir Y, Barberà JA, Champion H, Coghlan JG, Cottin V, De Marco T, Galiè N, Ghio S, Gibbs S, Martinez FJ, Semigran MJ, Simonneau G, Wells AU, Vachiéry J-L (2013). Pulmonary hypertension in chronic lung diseases. J Am Coll Cardiol.

[73] Jacob J, Bartholmai BJ, Rajagopalan S, Kokosi M, Maher TM, Nair A, Karwoski R, Renzoni E, Walsh SLF, Hansell DM, Wells AU (2017). Functional and prognostic effects when emphysema complicates idiopathic pulmonary fibrosis. Eur Respir J.

[74] Cottin Vincent, Azuma Arata, Raghu Ganesh, Stansen Wibke, Stowasser Susanne, Schlenker-Herceg Rozsa, Kolb Martin (2019). Therapeutic effects of nintedanib are not influenced by emphysema in the INPULSIS trials. European Respiratory Journal.

[75] Laporta Hernandez R, Aguilar Perez M, Lázaro Carrasco MT, Ussetti GP (2018). Lung transplantation in idiopathic pulmonary fibrosis. Med Sci.

[76] Weill D, Benden C, Corris PA, Dark JH, Davis RD, Keshavjee S, Lederer DJ, Mulligan MJ, Patterson GA, Singer LG, Snell GI, Verleden GM, Zamora MR, Glanville AR (2015). A consensus document for the selection of lung transplant candidates: 2014–An update from the Pulmonary Transplantation Council of the International Society for Heart and Lung Transplantation. J Heart Lung Transplant.

[77] Roth GJ, Binder R, Colbatzky F, Dallinger C, Schlenker-Herceg R, Hilberg F, Wollin S-L, Kaiser R (2015). Nintedanib: From discovery to the clinic. J Med Chem..

[78] Roodhart JM, Langenberg MH, Witteveen E, Voest EE (2008). The molecular basis of class side effects due to treatment with inhibitors of the VEGF/VEGFR pathway. Curr Clin Pharmacol.

[79] Shah RR, Morganroth J (2015). Update on cardiovascular safety of tyrosine kinase inhibitors: With a special focus on qt interval, left ventricular dysfunction and overall risk/benefit. Drug Saf..

[80] van Cruijsen H, van der Veldt A, Hoekman K (2009). Tyrosine kinase inhibitors of VEGF receptors: clinical issues and remaining questions. Front Biosci..

[81] Shah Devron R., Dholakia Shamik, Shah Rashmi R. (2014). Effect of Tyrosine Kinase Inhibitors on Wound Healing and Tissue Repair: Implications for Surgery in Cancer Patients. Drug Safety.

[82] Powers CJ, McLeskey SW, Wellstein A (2000). Fibroblast growth factors, their receptors and signaling. Endocr Relat Cancer.

[83] Noth Imre, Oelberg David, Kaul Manika, Conoscenti Craig S., Raghu Ganesh (2018). Safety and tolerability of nintedanib in patients with idiopathic pulmonary fibrosis in the USA. European Respiratory Journal.

[84] Hughes G, Toellner H, Morris H, Leonard C, Chaudhuri N (2016). Real world experiences: Pirfenidone and nintedanib are effective and well tolerated treatments for idiopathic pulmonary fibrosis. J Clin Med.

[85] Bonella F, Kreuter M, Hagmeyer L, Neurohr C, Keller C, Kohlhaeufl MJ, Müller-Quernheim J, Milger K, Prasse A (2016). Insights from the German compassionate use program of nintedanib for the treatment of idiopathic pulmonary fibrosis. Respiration..

[86] Tzouvelekis A, Karampitsakos T, Kontou M, Granitsas A, Malliou I, Anagnostopoulos A, Ntolios P, Tzilas V, Bouros E, Steiropoulos P, Chrysikos S, Dimakou K, Koulouris N, Bouros D (2018). Safety and efficacy of nintedanib in idiopathic pulmonary fibrosis: A real-life observational study in Greece. Pulm Pharmacol Ther.

[87] Barratt SL, Mulholland S, Al Jbour K, Steer H, Gutsche M, Foley N, Srivastava R, Sharp C, Adamali HI (2018). South–west of England’s experience of the safety and tolerability pirfenidone and nintedanib for the treatment of idiopathic pulmonary fibrosis (IPF). Front Pharmacol.

[88] Bendstrup E, Wuyts W, Alfaro T, Chaudhuri N, Cornelissen R, Kreuter M, Melgaard Nielsen K, Münster AMB, Myllärniemi M, Ravaglia C, Vanuytsel T, Wijsenbeek M (2019). Nintedanib in idiopathic pulmonary fibrosis: Practical management recommendations for potential adverse events. Respiration.

[89] Delanote I, Wuyts WA, Yserbyt J, Verbeken EK, Verleden GM, Vos R (2016). Safety and efficacy of bridging to lung transplantation with antifibrotic drugs in idiopathic pulmonary fibrosis: a case series. BMC Pulm Med.

[90] Leuschner G, Stocker F, Veit T, Kneidinger N, Winter H, Schramm R, Weig T, Matthes S, Ceelen F, Arnold P, Munker D, Klenner F, Hatz R, Frankenberger M, Behr J, Neurohr C (2018). Outcome of lung transplantation in idiopathic pulmonary fibrosis with previous anti-fibrotic therapy. J Heart Lung Transplant.

[91] Lambers C, Boehm PM, Lee S, Ius F, Jaksch P, Klepetko W, Tudorache I, Ristl R, Welte T, Gottlieb J (2018). Effect of antifibrotics on short-term outcome after bilateral lung transplantation: a multicentre analysis. Eur Respir J.

[92] Turner-Warwick M, Lebowitz M, Burrows B, Johnson A (1980). Cryptogenic fibrosing alveolitis and lung cancer. Thorax.

[93] Yoon JH, Nouraie M, Chen X, Zou RH, Sellares J, Veraldi KL, Chiarchiaro J, Lindell K, Wilson DO, Kaminski N, Burns T, Trejo Bittar H, Yousem S, Gibson K, Kass DJ (2018). Characteristics of lung cancer among patients with idiopathic pulmonary fibrosis and interstitial lung disease – analysis of institutional and population data. Respir Res.

[94] Karampitsakos T, Tzilas V, Tringidou R, Steiropoulos P, Aidinis V, Papiris SA, Bouros D, Tzouvelekis A (2017). Lung cancer in patients with idiopathic pulmonary fibrosis. Pulm Pharmacol Ther.

[95] Raghu G, Amatto VC, Behr J, Stowasser S (2015). Comorbidities in idiopathic pulmonary fibrosis patients: a systematic literature review. Eur Respir J.

[96] Kreuter M, Ehlers-Tenenbaum S, Schaaf M, Oltmanns U, Palmowski K, Hoffmann H, Schnabel PA, Heussel CP, Puderbach M, Herth FJ, Warth A (2015). Treatment and outcome of lung cancer in idiopathic interstitial pneumonias. Sarcoidosis Vasc Diffuse Lung Dis..

[97] Vargatef. Summary of product characteristics: Boehringer Ingelheim; 2018. https://www.medicines.org.uk/emc/product/3647/smpc. Accessed 25 Feb 2019

[98] Dhillon S (2015). Nintedanib: A review of its use as second-line treatment in adults with advanced non-small cell lung cancer of adenocarcinoma histology. Target Oncol..

[99] Oldham JM, Collard HR (2017). Comorbid conditions in idiopathic pulmonary fibrosis: recognition and management. Front Med.

[100] Fukunaga K, Yokoe S, Kawashima S, Uchida Y, Nakagawa H, Nakano Y (2018). Nintedanib prevented fibrosis progression and lung cancer growth in idiopathic pulmonary fibrosis. Respirol Case Rep.

[101] Otsubo K, Kishimoto J, Kenmotsu H, Minegishi Y, Ichihara E, Shiraki A, Kato T, Atagi S, Horinouchi H, Ando M, Kondoh Y, Kusumoto M, Ichikado K, Yamamoto N, Nakanishi Y, Okamoto I (2018). Treatment rationale and design for J-SONIC: A randomized study of carboplatin plus nab-paclitaxel with or without nintedanib for advanced non-small-cell lung cancer with idiopathic pulmonary fibrosis. Clin Lung Cancer.

[102] Richeldi L, Kreuter M, Selman M, Crestani B, Kirsten A-M, Wuyts WA, Xu Z, Bernois K, Stowasser S, Quaresma M, Costabel U (2018). Long-term treatment of patients with idiopathic pulmonary fibrosis with nintedanib: results from the TOMORROW trial and its open-label extension. Thorax..

[103] Corte T, Bonella F, Crestani B, Demedts MG, Richeldi L, Coeck C, Pelling K, Quaresma M, Lasky JA (2015). Safety, tolerability and appropriate use of nintedanib in idiopathic pulmonary fibrosis. Respir Res.

[104] Distler O, Highland KB, Gahlemann M, Azuma A, Fischer A, Mayes MD, Raghu G, Sauter W, Girard M, Alves M, Clerisme-Beaty E, Stowasser S, Tetzlaff K, Kuwana M, Maher TM (2019). Nintedanib for systemic sclerosis–associated interstitial lung disease. N Engl J Med.

[105] Flaherty KR, Wells AU, Clerisme-Beaty E, Cottin V, Devaraj A, Inoue Y, Richeldi L, Walsh S, Goeldner R-G, Schlenker-Herceg R, Brown KK, investigators obotIt. Characteristics of patients with progressive fibrosing interstitial lung diseases (ILDs) in the INBUILD trial of nintedanib. Am J Respir Crit Care Med. 2019;199:A5627.

[106] Arthritis advisory committee briefing materials (2019). Nintedanib soft capsules.

[107] Harari S, Caminati A, Poletti V, Confalonieri M, Gasparini S, Lacedonia D, Luppi F, Pesci A, Sebastiani A, Spagnolo P, Vancheri C, Balestro E, Bonifazi M, Cerri S, De Giacomi F, Della Porta R, Foschino Barbaro MP, Fui A, Pasquinelli P, Rosso R, Tomassetti S, Specchia C, Rottoli P (2018). A real-life multicenter national study on nintedanib in severe idiopathic pulmonary fibrosis. Respiration.

[108] Bargagli E, Piccioli C, Rosi E, Torricelli E, Turi L, Piccioli E, Pistolesi M, Ferrari K, Voltolini L (2019). Pirfenidone and nintedanib in idiopathic pulmonary fibrosis: Real-life experience in an Italian referral centre. Pulmonology.

[109] Toellner H, Hughes G, Beswick W, Crooks MG, Donaldson C, Forrest I, Hart SP, Leonard C, Major M, Simpson AJ, Chaudhuri N (2017). Early clinical experiences with nintedanib in three UK tertiary interstitial lung disease centres. Clin Transl Med.

[110] Brunnemer E, Wälscher J, Tenenbaum S, Hausmanns J, Schulze K, Seiter M, Heussel CP, Warth A, Herth FJF, Kreuter M (2018). Real-world experience with nintedanib in patients with idiopathic pulmonary fibrosis. Respiration.

[111] Maher T, Noth I, Allinger A, Kaul M, Conoscenti C, Oelberg D (2016). P168 Safety and tolerability of nintedanib in patients with idiopathic pulmonary fibrosis (IPF): One-year data from post-marketing surveillance in the United States. Thorax.

[112] Kreuter M, Ehlers-Tenenbaum S, Palmowski K, Bruhwyler J, Oltmanns U, Muley T, Heussel CP, Warth A, Kolb M, Herth FJF (2016). Impact of comorbidities on mortality in patients with idiopathic pulmonary fibrosis. PLoS One.

[113] Kreuter M, Swigris J, Pittrow D, Geier S, Klotsche J, Prasse A, Wirtz H, Koschel D, Andreas S, Claussen M, Grohé C, Wilkens H, Hagmeyer L, Skowasch D, Meyer JF, Kirschner J, Gläser S, Herth FJF, Welte T, Neurohr C, Schwaiblmair M, Held M, Bahmer T, Frankenberger M, Behr J (2017). Health related quality of life in patients with idiopathic pulmonary fibrosis in clinical practice: insights-IPF registry. Respir Res.

[114] Tomioka H, Takada H (2017). Treatment with nintedanib for acute exacerbation of idiopathic pulmonary fibrosis. Respirol Case Rep.

[115] Ito Y, Tazaki G, Kondo Y, Takahashi G, Sakamaki F (2019). Therapeutic effect of nintedanib on acute exacerbation of interstitial lung diseases. Respir Med Case Rep.

[116] Jo HE, Glaspole I, Grainge C, Goh N, Hopkins PM, Moodley Y, Reynolds PN, Chapman S, Walters EH, Zappala C, Allan H, Keir GJ, Hayen A, Cooper WA, Mahar AM, Ellis S, Macansh S, Corte TJ (2017). Baseline characteristics of idiopathic pulmonary fibrosis: analysis from the Australian Idiopathic Pulmonary Fibrosis Registry. Eur Respir J..

[117] Behr J, Wirtz H, Pittrow D, Prasse A, Koschel D, Geier S, Klotsche J, Andreas S, Claussen M, Grohé C, Wilkens H, Hagmeyer L, Skowasch D, Meyer JF, Kirschner J, Gläser S, Kahn N, Welte T, Neurohr C, Schwaiblmair M, Held M, Bahmer T, Oqueka T, Frankenberger M, Kreuter M (2019). Survival and course of lung function in patients with idiopathic pulmonary fibrosis with or without antifibrotic treatment: long-term results of the INSIGHTS-IPF registry.

[118] Ogura T, Taniguchi H, Azuma A, Inoue Y, Kondoh Y, Hasegawa Y, Bando M, Abe S, Mochizuki Y, Chida K, Klüglich M, Fujimoto T, Okazaki K, Tadayasu Y, Sakamoto W, Sugiyama Y (2015). Safety and pharmacokinetics of nintedanib and pirfenidone in idiopathic pulmonary fibrosis. Eur Respir J..

[119] Flaherty KR, Fell CD, Huggins JT, Nunes H, Sussman R, Valenzuela C, Petzinger U, Stauffer JL, Gilberg F, Bengus M, Wijsenbeek M (2018). Safety of nintedanib added to pirfenidone treatment for idiopathic pulmonary fibrosis. Eur Respir J..

[120] Milger K, Kneidinger N, Neurohr C, Reichenberger F, Behr J (2015). Switching to nintedanib after discontinuation of pirfenidone due to adverse events in IPF. Eur Respir J.

[121] Lee JS, Collard HR, Anstrom KJ, Martinez FJ, Noth I, Roberts RS, Yow E, Raghu G (2013). Anti-acid treatment and disease progression in idiopathic pulmonary fibrosis: an analysis of data from three randomised controlled trials. Lancet Respir Med.

[122] Kreuter M, Wuyts W, Renzoni E, Koschel D, Maher TM, Kolb M, Weycker D, Spagnolo P, Kirchgaessler K-U, Herth FJF, Costabel U (2016). Antacid therapy and disease outcomes in idiopathic pulmonary fibrosis: a pooled analysis. Lancet Respir Med.

[123] Costabel U, Behr J, Crestani B, Stansen W, Schlenker-Herceg R, Stowasser S, Raghu G (2018). Anti-acid therapy in idiopathic pulmonary fibrosis: insights from the INPULSIS® trials. Respir Res.

[124] Bavishi C, DuPont HL (2011). Systematic review: The use of proton pump inhibitors and increased susceptibility to enteric infection. Aliment Pharmacol Ther.

[125] Somogyi Vivien, Chaudhuri Nazia, Torrisi Sebastiano Emanuele, Kahn Nicolas, Müller Veronika, Kreuter Michael (2019). The therapy of idiopathic pulmonary fibrosis: what is next?. European Respiratory Review.

[126] Maher TM, Kreuter M, Lederer DJ, Brown KK, Wuyts W, Verbruggen N, Stutvoet S, Fieuw A, Ford P, Abi-Saab W, Wijsenbeek M (2019). Rationale, design and objectives of two phase III, randomised, placebo-controlled studies of GLPG1690, a novel autotaxin inhibitor, in idiopathic pulmonary fibrosis (ISABELA 1 and 2). BMJ Open Respir Res.

[127] Raghu G, van den Blink B, Hamblin MJ, Brown AW, Golden JA, Ho LA, Wijsenbeek MS, Vasakova M, Pesci A, Antin-Ozerkis DE, Meyer KC, Kreuter M, Santin-Janin H, Mulder G-J, Bartholmai B, Gupta R, Richeldi L (2018). Effect of recombinant human pentraxin 2 vs placebo on change in forced vital capacity in patients with idiopathic pulmonary fibrosis: A randomized clinical trialrecombinant human pentraxin 2 vs placebo and lung function in idiopathic pulmonary fibrosisrecombinant human pentraxin 2 vs placebo and lung function in idiopathic pulmonary fibrosis. J Am Med Assoc.

[128] Khalil Nasreen, Manganas Helene, Ryerson Christopher J., Shapera Shane, Cantin Andre M., Hernandez Paul, Turcotte Eric E., Parker Joseph M., Moran John E., Albert Gary R., Sawtell Renata, Hagerimana Aline, Laurin Pierre, Gagnon Lyne, Cesari Frank, Kolb Martin (2018). Phase 2 clinical trial of PBI-4050 in patients with idiopathic pulmonary fibrosis. European Respiratory Journal.

[129] Gorina E, Richeldi L, Raghu G, Fernandez Perez E, Costabel U, Albera C, Lederer D, Flaherty K, Ettinger N, Bercz P, Singh B, Perez R, Goldin J, Kouchakji E, Porter S (2017). PRAISE, a randomized, placebo-controlled, double-blind Phase 2 clinical trial of pamrevlumab (FG-3019) in IPF patients. Eur Respir J..

[130] Dowman L, Hill CJ, Holland AE. Pulmonary rehabilitation for interstitial lung disease. Cochrane Database Syst Rev. 2014;(10):CD006322. 10.1002/14651858.CD006322.pub3 25284270

[131] Study of pulmonary rehabilitation in patients with idiopathic pulmonary fibrosis (IPF). 2019. https://clinicaltrials.gov/ct2/show/NCT03717012. Accessed 12 Apr 2019.

[132] White ES, Borok Z, Brown KK, Eickelberg O, Guenther A, Jenkins RG, Kolb M, Martinez FJ, Roman J, Sime P, American Thoracic Society Respiratory C, Molecular Biology Assembly Working Group on Pulmonary F (2016). An American Thoracic Society official research statement: Future directions in lung fibrosis research. Am J Respir Crit Care Med.

[133] Wells AU, Brown KK, Flaherty KR, Kolb M, Thannickal VJ (2018). What's in a name? That which we call IPF, by any other name would act the same. Eur Respir J.

[134] Cottin V, Hirani NA, Hotchkin DL, Nambiar AM, Ogura T, Otaola M, Skowasch D, Park JS, Poonyagariyagorn HK, Wuyts W, Wells AU (2018). Presentation, diagnosis and clinical course of the spectrum of progressive-fibrosing interstitial lung diseases. Eur Respir Rev.

[135] Richeldi L, Varone F, Bergna M, de Andrade J, Falk J, Hallowell R, Jouneau S, Kondoh Y, Morrow L, Randerath W, Strek M, Tabaj G (2018). Pharmacological management of progressive-fibrosing interstitial lung diseases: a review of the current evidence. Eur Respir Rev.

[136] Cottin V (2019). Treatment of progressive fibrosing interstitial lung diseases: a milestone in the management of interstitial lung diseases. Eur Respir Rev..

[137] Tyndall AJ, Bannert B, Vonk M, Airò P, Cozzi F, Carreira PE, Bancel DF, Allanore Y, Müller-Ladner U, Distler O, Iannone F, Pellerito R, Pileckyte M, Miniati I, Ananieva L, Gurman AB, Damjanov N, Mueller A, Valentini G, Riemekasten G, Tikly M, Hummers L, Henriques MJ, Caramaschi P, Scheja A, Rozman B, Ton E, Kumánovics G, Coleiro B, Feierl E, Szucs G, Von Mühlen CA, Riccieri V, Novak S, Chizzolini C, Kotulska A, Denton C, Coelho PC, Kötter I, Simsek I, de la Pena Lefebvre PG, Hachulla E, Seibold JR, Rednic S, Štork J, Morovic-Vergles J, Walker UA (2010). Causes and risk factors for death in systemic sclerosis: a study from the EULAR Scleroderma Trials and Research (EUSTAR) database. Ann Rheum Dis.

[138] Bergamasco A, Hartmann N, Wallace L, Verpillat P (2019). Epidemiology of systemic sclerosis and systemic sclerosis-associated interstitial lung disease. Clin Epidemiol..

[139] Cottin V, Brown KK (2019). Interstitial lung disease associated with systemic sclerosis (SSc-ILD). Respir Res.

[140] Goh NS, Hoyles RK, Denton CP, Hansell DM, Renzoni EA, Maher TM, Nicholson AG, Wells AU (2017). Short-term pulmonary function trends are predictive of mortality in interstitial lung disease associated with systemic sclerosis. Arthritis Rheumatol.

[141] Man A, Davidyock T, Ferguson LT, Ieong M, Zhang Y, Simms RW (2015). Changes in forced vital capacity over time in systemic sclerosis: application of group-based trajectory modelling. Rheumatology (Oxford).

[142] Flaherty Kevin R., Wells Athol U., Cottin Vincent, Devaraj Anand, Walsh Simon L.F., Inoue Yoshikazu, Richeldi Luca, Kolb Martin, Tetzlaff Kay, Stowasser Susanne, Coeck Carl, Clerisme-Beaty Emmanuelle, Rosenstock Bernd, Quaresma Manuel, Haeufel Thomas, Goeldner Rainer-Georg, Schlenker-Herceg Rozsa, Brown Kevin K. (2019). Nintedanib in Progressive Fibrosing Interstitial Lung Diseases. New England Journal of Medicine.

[143] Goldberg Hilary J. (2019). Understanding Progressive Fibrosing Interstitial Lung Disease through Therapeutic Trials. New England Journal of Medicine.

[144] Torrisi SE, Kahn N, Wälscher J, Sarmand N, Polke M, Lars K, Eichinger M, Heussel CP, Palmucci S, Sambataro FM, Sambataro G, Sambataro D, Vancheri C, Kreuter M (2019). Possible value of antifibrotic drugs in patients with progressive fibrosing non-IPF interstitial lung diseases. BMC Pulm Med..

